# Cross-platform ultradeep transcriptomic profiling of human reference RNA samples by RNA-Seq

**DOI:** 10.1038/sdata.2014.20

**Published:** 2014-08-26

**Authors:** Joshua Xu, Zhenqiang Su, Huixiao Hong, Jean Thierry-Mieg, Danielle Thierry-Mieg, David P. Kreil, Christopher E. Mason, Weida Tong, Leming Shi

**Affiliations:** 1 Division of Bioinformatics and Biostatistics, National Center for Toxicological Research, Food and Drug Administration, Jefferson, Arkansas 72079, USA; 2 National Center for Biotechnology Information, National Library of Medicine, National Institutes of Health, Bethesda, Maryland 20814, USA; 3 Chair of Bioinformatics Research Group, Boku University Vienna, Vienna, Austria; 4 University of Warwick, Coventry CV4 7AL, UK; 5 Department of Physiology and Biophysics and the Institute for Computational Biomedicine, Weill Cornell Medical College, New York, New York 10021, USA; 6 State Key Laboratory of Genetic Engineering and MOE Key Laboratory of Contemporary Anthropology, Schools of Life Sciences and Pharmacy, Fudan University, Shanghai 201203, China

## Abstract

Whole-transcriptome sequencing (‘RNA-Seq’) has been drastically changing the scale and scope of genomic research. In order to fully understand the power and limitations of this technology, the US Food and Drug Administration (FDA) launched the third phase of the MicroArray Quality Control (MAQC-III) project, also known as the SEquencing Quality Control (SEQC) project. Using two well-established human reference RNA samples from the first phase of the MAQC project, three sequencing platforms were tested across more than ten sites with built-in truths including spike-in of external RNA controls (ERCC), titration data and qPCR verification. The SEQC project generated over 30 billion sequence reads representing the largest RNA-Seq data ever generated by a single project on individual RNA samples. This extraordinarily ultradeep transcriptomic data set and the known truths built into the study design provide many opportunities for further research and development to advance the improvement and application of RNA-Seq.

## Background & Summary

The recent advancement of next-generation sequencing (NGS) has generated tremendous opportunities and challenges in the communities of biomedical research, public health genomics and personalized medicine. Among the versatile applications of NGS, whole-transcriptome sequencing (‘RNA-Seq’ or WTS) has enabled quantitative profiling with a large dynamic range^1^. As demonstrated in many publications, RNA-Seq enables the discovery of new structural elements of genes such as exons, junctions, untranslated regions, and rare isoforms and thus has expanded our understanding of the transcriptome^2–5,2–5,2–5,2–5^. It provides increased sensitivity compared to the more mature microarray technology^6^ and has opened new avenues of research in transcriptome work, such as the study of gene fusions and allele-specific expression, or the discovery of novel alternative transcripts, whereas the measurement noise of RNA-Seq was shown to be a direct consequence of the random sampling process^7–9,7–9,7–9^.

While new platforms and protocols for RNA-Seq have emerged in recent years, the comparability of results across platforms and laboratories has not been extensively examined. With the widespread adoption of RNA-Seq in biomedical and clinical research, a comprehensive, cross-site and cross-platform analysis of the performance of RNA-Seq is essential. Reproducibility across laboratories, in particular, is a crucial requirement for any new experimental method to be relevant for research and clinical applications, and this can only be tested in an extensive multi-site and multi-platform comparison. Just as in the first phase of the MicroArray Quality Control (MAQC-I) project^10^, which tested multi-site and multi-platform agreement in gene-expression microarrays, the FDA coordinated again the third phase of MAQC (MAQC-III) as a large-scale community effort to assess the performance of RNA-Seq, testing different sequencing platforms and analysis pipelines. This project is also known as the SEquencing Quality Control (SEQC) project. A complementary effort that utilized the same samples, but different platforms (e.g., Life Technologies’ Ion PGM and Ion Proton, and Pacific Biosciences’ PacBio RS) and library protocols (polyA selection, ribosome depletion, size-selection, and RNA degradation) was coordinated with the Association of Biomolecular Resource Facilities (ABRF) Next Generation Sequencing Study (ABRF-NGS)^11^.

**Figure 1:**
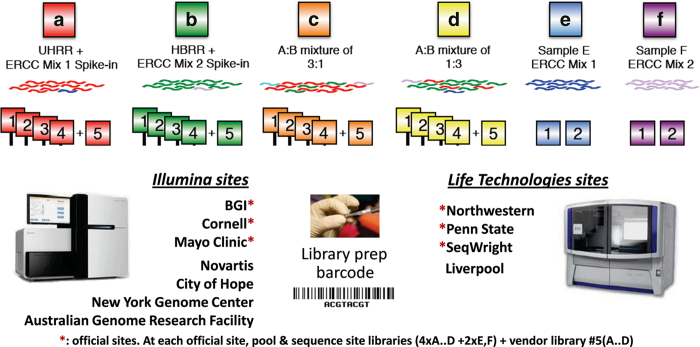
SEQC study design. This figure was modified from **b** presented in the related research manuscript^13^. Similar to the MAQC-I benchmarks, well characterized RNA samples A and B were augmented by samples C and D comprised of A and B in known mixing ratios 3:1 and 1:3, respectively. These allow tests for titration consistency and the correct recovery of the known mixing ratios. Synthetic RNAs from the External RNA Control Consortium (ERCC) were both pre-added to samples A and B before mixing and also sequenced separately to assess dynamic range (samples E and F). Samples were distributed to independent sites for RNA-Seq library construction and profiling by Illumina’s HiSeq 2000 (3+4x) and Life Technologies’ SOLiD 5500 (3+1x). In addition to the replicate libraries A1…D4 at each site, for each platform, one vendor-prepared library A5…D5 was being sequenced at all three official sites, giving a total of 24 libraries. At each site, each library has a unique barcode sequence and all libraries were pooled before sequencing, so each lane was sequencing the same material, allowing a study of lane specific effects. Samples A and B were also sequenced by Roche 454 GS FLX at different sites with two runs each but no library replicates.

**Figure 2:**
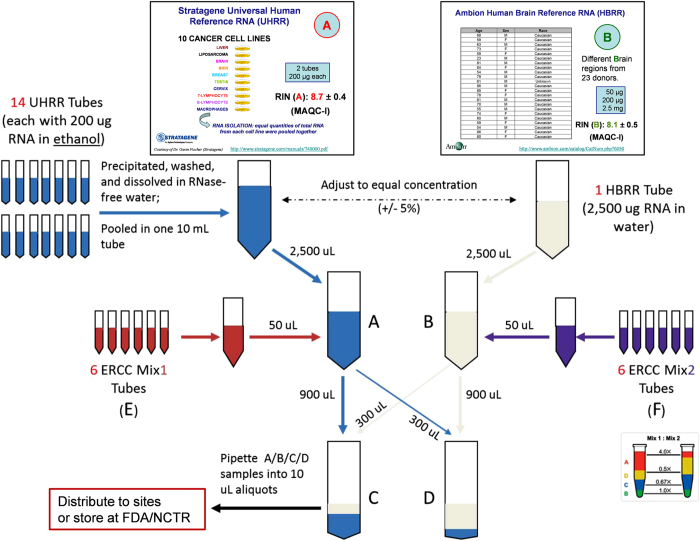
Mixing scheme to generate the SEQC RNA samples. This figure was modified from Supplementary Figure S1 presented in the related research manuscript^13^. Samples MAQC-I–*A* and MAQC-I–*B* (top) were thawed from the stock acquired during the original MAQC-I study (2006) and aliquots then pooled (blue and grey tubes), adjusted to equal concentration, and then mixed with ERCC mix sets *E* and *F* (respectively). The ERCC mixes contain 4 subgroups of transcripts with different molar concentration ratios (4.0, 0.67, 0.5, and 1.0) defined between the two mixes (right bottom). Equal portions of these mixtures were then titrated in 3:1 and 1:3 ratios to create samples *C* and *D* (bottom). All four samples were finally separated into 10 μl aliquots for storage and distribution to the sequencing sites.

The objective assessment of technical performance such as accuracy and sensitivity is a great challenge since there is no independent ‘gold standard’. In the SEQC study, such assessments were achieved in a controlled test setting, where truths built into the study design could be directly validated, and then related to the performance of other transcriptome profiling technologies. Specifically, we utilized the two well-characterized human reference RNA samples A (Universal Human Reference RNA) and B (Human Brain Reference RNA) from the MAQC consortium, which had been studied extensively with microarrays in MAQC-I^10^. With spike-ins of synthetic RNA from the External RNA Control Consortium (ERCC)^12^, samples A and B were then mixed to construct samples C and D in known mixing ratios, 3:1 and 1:3, respectively (Figures 1 and 2). All samples were distributed to independent sites for RNA-Seq library construction and profiling by Illumina’s HiSeq 2000 platform (7 sites) and Life Technologies’ SOLiD 5500 platform (4 sites). In addition, vendors created their own cDNA libraries that were then distributed to each test site, in order to examine the degree of a ‘site effect’ that was independent of the library preparation process (Figure 1). As depicted in Figure 1, numbers 1–4 denote the 4 libraries prepared by the test sites themselves, while number 5 indicates the library created by the vendors. To support an assessment of gene models, samples A and B were also sequenced at three independent sites by the Roche 454 GS FLX platform, providing longer reads. For comparison to other technologies, data were also compared to the GeneChip Human Genome U133 Plus 2.0 microarrays used in MAQC-I, several current microarray platforms, and also assessed by 20,801 PrimePCR reactions^13^ and a set of TaqMan assays from MAQC-I^14^. These data create an overlapping framework of orthogonal validation for any expression measure, splice form, or gene structure question.

Thus different sequencing platforms were tested using four well-characterized reference RNA sample mixtures with built-in truths to test accuracy, precision, reproducibility, sensitivity and specificity in a detailed analysis of over 30 billion reads on these reference samples (Table 1). The data presented here provide the deepest molecular characterization of any RNA samples published to date. Leveraging this ultradeep transcriptomic data set and the known truths built into the study design, in our related work^13^, we provided an in depth analysis of these data and found that RNA-Seq was highly reproducible across sites and platforms, particularly in differential gene-expression analysis. However, performance was clearly dependent on data treatment and analysis, and transcript-level profiling showed larger variation. This indicates ample opportunities offered by this unique data set: algorithms and pipelines with better and more consistent performance may be developed for transcripts assembly and quantification, gene expression quantification, and gene fusion detection. The presented data set can thus serve a key resource in the development and validation of novel RNA-Seq data analysis algorithms to advance the maturity and performance of applications of RNA-Seq. In this Data Descriptor, we provide additional information aimed at helping others reuse these data within their own research, including more detailed methods descriptions.

## Methods

### RNA sample preparation

This description on RNA sample preparation is expanded from descriptions in the related research manuscript^13^. The SEQC (MAQC-III) study design is based on the well-characterized MAQC-I RNA samples: the Universal Human Reference RNA (UHRR, from 10 pooled cancer cell lines, Agilent Technologies, Inc.) and the Human Brain Reference RNA (HBRR, from multiple brain regions of 23 donors, Life Technologies, Inc.)^10^. More details about these two RNA reference samples were shown in Figure 2. To these, two different ERCC^12^ spike-in mixes (Life Technologies, Inc.) were added (50 μl of ERCC mix was spiked into 2,500 μl sample with a total RNA concentration around 1 μg/μl) to give: Sample A—UHRR with ERCC spike-in mix #1 (Sample E), and Sample B–HBRR with ERCC spike-in mix #2 (Sample F), following the manufacturer's protocol. The ERCC mixes contain 4 subgroups of transcripts with different molar concentration ratios defined between the two mixes (Figure 2). Equal amounts of Samples A and B (1,200 μl each) were then combined in ratios of 3:1 and 1:3, respectively, to generate equal amounts of Samples C and D (1,200 μl each) (Figure 2). Equal amounts of the samples A, B, C and D (10 μl) were aliquoted for storage at the FDA's National Center for Toxicological Research for distribution to sequencing sites. In total, 510 aliquots of the samples were produced. Once receiving the RNA samples, each sequencing site and the platform vendor verified the RNA quality with an Agilent Bioanalyzer 2100 (RIN>7.4).

### RNA-Seq sequencing sites

These descriptions on RNA-Seq sequencing sites are expanded from descriptions in the related research manuscript^13^. Each sequencing site was assigned a three-letter code and each platform vendor designated three ‘official sites’ (superscripted by *) before samples were distributed. Illumina HiSeq 2000 data were provided by 7 sites (ordered alphabetically by the site code): (1) Australian Genome Research Facility (AGR); (2) Beijing Genomics Institute (BGI)*; (3) Weill Cornell Medical College (CNL)*; (4) City of Hope (COH); (5) Mayo Clinic (MAY)*; (6) Novartis (NVS); and (7) the New York Genome Center (NYG), generating 100+100 nt read-pairs. Life Technologies SOLiD 5500 data were provided by 4 sites: (1) the University of Liverpool (LIV); (2) Northwestern University (NWU)*; (3) the Pennsylvania State University (PSU)*; and (4) SeqWright Inc. (SQW)*, generating 51+36 nt read-pairs, except for Liverpool which applied a protocol variant giving single 76 nt reads.

All official sites created 4 replicate measurements (libraries) of each sample A to D (labeled as 1–4 in Figure 1), and also sequenced a vendor-prepared fifth replicate (labeled as 5 in Figure 1). The non-official HiSeq 2000 sites sequenced only 4 replicate libraries of each sample A to D. In Liverpool, only one site-prepared library and one vendor-provided library of each of the samples A to D were sequenced. Data from all sites were distributed to all analysis sites of the SEQC consortium and later deposited to a public data repository (Data Citation 1). Data produced by the official sites were used in all of the analyses reported in the related research manuscript^13^. In addition, data produced by the non-official sites were incorporated in some analyses, e.g., the analysis of gene detection and junction discovery as a function of read depth^13^.

Roche 454 GS FLX data were provided by: (1) the Medical Genomes Project (MGP); (2) the New York University Medical Center (NYU); and (3) SeqWright Inc. (SQW). At each site, one replicate of samples A and B was sequenced (two runs). Roche 454 sequencing data were used to assess gene models but not for quantitative evaluation.

### Illumina RNA-Seq library preparation and sequencing

A workgroup was formed with representatives from Illumina and the three official Illumina sequencing sites to agree on an SEQC-specific sequencing SOP (standard operating procedure) based on the low-throughput protocol laid out in the Illumina TruSeq RNA Sample Prep Guide. Briefly, 250 ng of each total RNA sample was used for polyA mRNA selection and fragmentation; followed by first and second strand synthesis, end repair, adenylation of 3′ ends, and barcoded adapter ligation. Thus each library was made from an independent polyA mRNA selection. Each library was enriched by 15 cycles of PCR and the size distribution was validated on the Agilent Bioanalyzer using a DNA 1000 kit. The libraries were made from a band between 200–500 bp with a peak at approximately 275 bp. The cDNA libraries (and vendor prepared libraries for the three official sequencing sites) were each normalized to 10 nM and then pooled. The template cDNA was then diluted to a concentration of 10 pM prior to cluster generation. Equal amounts of pooled libraries were then loaded to each lane of 2 flow cells and run on HiSeq 2000 Sequencing Systems (Illumina) for paired-end 200 cycles. The Illumina sites produced on average 110 million read-pairs *per* replicate, for a total of 2,200 million per site (Table 1).

### SOLiD RNA-Seq library preparation and sequencing

Similar to the Illumina platform, a workgroup was also formed with representatives from the three official SOLiD sequencing sites to reach consensus on an SEQC-specific sequencing SOP (standard operating procedure) based on the low input protocol of the manufacturer's SOLiD Total RNA-Seq Kit Protocol (Life Technologies, Inc.). Due to the sample input requirement of Poly(A)Purist MAG kit (Life Technologies, Inc.), two rounds of polyA selection were performed with 50 μg of total RNA from each type of samples A–D. The yield and quality of the polyA mRNA were assessed using the Agilent 2100 Bioanalyzer. Four replicate libraries were prepared with each starting from 25 ng of polyA mRNA, following these major steps: RNA fragmentation, hybridization and ligation, reverse transcription, purification and size selection, barcoding and amplification, and purification. The yield and size distribution of the barcoded libraries were assessed and then pooled with vendor prepared libraries, followed by EZ bead emulsification at the E120 scale, amplification, and enrichment. The beads were deposited on the flow chip and sequenced 51×36 cycles on a SOLiD 5500XL sequencer. The official SOLiD sites produced on average 50 million read-pairs *per* replicate, for a total of 980 million per site (Table 1). The Liverpool site used exact call chemistry (ECC) reagents and generated 545 million single end reads (Table 1). ECC was reported to increase the accuracy of the SOLiD platform^15^.

### Roche 454 RNA-Seq library preparation and sequencing

One replicate library for sample A and B was prepared and sequenced on a Roche 454 GX FLX sequencer (two runs in total) at each site following the manufacturer's protocols. The Roche 454 sites produced on average 1 million reads per replicate, for a total of about 2.1million reads per site (Table 1).

### Data processing and naming convention

After base calling, adapter trimming, and barcode demultiplexing using the specific sequencer manufacturer's software, sequence data with quality scores were submitted from all sequencing sites to the FDA’s National Center for Toxicological Research (NCTR) that coordinated this project for integrity check, labeling check and reformatting. A naming convention was then applied to the uniformly reformatted data with a compact digital signature (MD5 checksum) computed for each data file. The name of each data file coded the following fields with valid values in parentheses: project (‘SEQC’), platform (‘ILM’ for Illumina HiSeq 2000, ‘LIF’ for Life Technologies SOLiD 5500, and ‘ROC’ for Roche 454 GS FLX), site (see the above section on RNA-Seq sequencing sites), RNA sample (‘A’–‘F’), replicate number (‘1’-‘5’), lane/sector number (‘L01’–‘L08’), indexing barcode (six letters of A/C/G/T for ILM or ten letters for LIF), flow-cell/flow-chip/slide ID, read direction defined by the platform (‘R1’ and ‘R2’ for ILM, ‘F3’ and ‘F5-RNA’ for LIF), and file type (‘fastq’ for ILM, ‘csfasta’ and ‘QV.qual’ for LIF, ‘fna’ and ‘qual’ for ROC). Applicable fields were concatenated in the above mentioned order with underscores (‘_’) as the field separators. Finally, each data file was compressed individually with gzip and attached with the suffix ‘gz’. Sequence data were then duplicated and distributed to data analysis sites of the SEQC consortium.

## Data Records

Gene Expression Omnibus (GEO) accession GSE47774 (Data Citation 1) contains files for the sequence read counts mapped to each of the human and ERCC transcripts. These files are in the following tab-separated format: NCBI RefSeq transcript ID and mapped reads count. Data Citation 1 also provides a link to the corresponding NCBI Sequence Read Archive (SRA) accession that contains the raw sequencing data from all sequencing sites. PrimePCR data and additional microarray data profiling the SEQC samples have also been deposited at GEO (Data Citation 2). All SEQC (MAQC-III) data sets are available through GEO (Data Citation 3). Data sets from the ABRF-NGS study using the same samples are also available through GEO (accession number: GSE46876). Microarray and qPCR data from the MAQC-I study are available through GEO (Data Citation 4).

## Technical Validation

The aim of QC and validation was to detect and correct any issues related to data integrity and data file labeling by taking advantage of the built-in truth in the study design. ERCC mixes 1 and 2 were spiked into samples A and B, respectively, prior to the mixing of samples A and B to make samples C and D. Furthermore, based on the pilot RNA-Seq data of samples A and B, a few genes had been identified to be highly abundant in one sample yet weakly expressed in the other. Ten of these genes were taken as sample-specific genes, five of which were highly expressed in sample A (with RefSeq transcript ID NM_000612.4, NM_001007139.4, NM_000384.2, NM_000477.5, and NR_003512.2) and five in sample B (with NCBI RefSeq accession NM_001025101.1, NM_001025092.1, NM_001025090.1, NM_002385.2, and NM_001025081.1). Mapping of reads to ERCC transcripts and these sample-specific genes was used to detect any deviation caused by data corruption or mislabeling from the built-in truth of ERCC spike-ins (about 1%) and sample titration. These mapping and quantification tools^16–18,16–18,16–18^ were used to build the QC pipeline: Bowtie v0.12.7 (ref. 16), samtools v0.1.18 (ref. 17), and Cufflinks v1.3.0 (ref. 18). The pooling of libraries also provided another quality check criterion, i.e., the proportion of each library per lane (in reads) being constant across all lanes for each sequencing site, as the pooled libraries were sequenced in all lanes of both flow-cells (Illumina) or flow-chips (SOLiD). This QC process identified mislabeling of about 1–3% data files submitted by some of the test sites. Consequently, we made sure that the sequence data were collected and labeled correctly and then transferred with high fidelity.

This multi-site study with replicated measurements at each site allows a comparison of the reproducibility of RNA-Seq between replicates intra-site and inter-site. Analysis results show that RNA-Seq is reproducible within sites, between sites, and across platforms for the detection of known genes and junctions, relative expression levels, and differential expression analysis. Furthermore, the built-in truth (i.e., sample titration and the external ERCC spike-in) allows a number of assays reflecting both accuracy and precision of relative quantitative measurements: (1) titration order consistency, (2) known sample mixture ratio recovery, and (3) recovery of ERCC transcript mixture ratios. We observed that the majority of genes (59%) correctly titrated, with little disagreement between platforms. And the correct ratio was recovered for the majority of genes, with better agreement at higher expression levels (top 25%). Across platforms, we also observed that with sufficiently high expression levels (log2[conc]>3), the expected ERCC ratios (Figure 2) of 1/2, 2/3, 1, and 4 were accurately recovered using about 90 million mapped fragments, with high precision indicating good reproducibility.

## Usage Notes

Many publicly available software packages^19^ could be used to analyze these RNA-Seq data. Using a variety of tools^13^, the SEQC consortium conducted a detailed analysis of this rich data set to test the performance of RNA-Seq. This extraordinarily ultradeep data set provides the deepest molecular characterization of any RNA samples published to date. With the known truths built into the study design, it provides ample opportunities for further research and development. For example, efficient quantitative expression profiling takes advantage of known gene models, and the choice of a reference annotation can considerably affect results, as reflected in performance assessments. Particularly quantitative expression profiling of alternative transcripts forms a promising area for future efforts^13^. The depth of this data and the included long reads from Roche 454 can further be utilized to enrich and refine gene models and annotation, which are critical for effective quantitative profiling.

## Additional information

**How to cite this article:** Xu, J. *et al.* Cross-platform ultradeep transcriptomic profiling of human reference RNA samples by RNA-Seq. *Sci. Data* 1:140020 doi: 10.1038/sdata.2014.20 (2014).

## Supplementary Material



## Figures and Tables

**Table 1:** Number of sequence reads (in millions) produced at each site, listed by sample and library replicate.

		**Illumina HiSeq 2000 sequencing sites**							**SOLiD 5500 sequencing sites**				**Roche 454 sequencing sites**		
**Sample**	**Replicate**	**BGI**	**CNL**	**MAY**	**AGR**	**COH**	**NVS**	**NYG**	**NWU**	**PSU**	**SQW**	**LIV**	**MGP**	**NYU**	**SQW**
A	1	189	201	139	318	201	333	76	123	105	118	91	0.57	0.55	0.50
A	2	157	184	256	343	202	382	79	124	115	130		0.43	0.61	0.49
A	3	188	134	199	297	201	358	68	105	141	87				
A	4	216	250	432	415	196	361	67	135	83	125				
A	5	191	144	111					53	34	67	58			
B	1	228	222	248	353	198	337	71	101	133	121	85	0.59	0.62	0.41
B	2	224	225	219	386	203	349	74	92	82	104		0.56	0.59	0.42
B	3	237	226	251	352	201	363	49	87	90	114				
B	4	175	188	258	329	192	370	76	148	67	91				
B	5	134	121	90					56	38	76	49			
C	1	183	226	154	412	209	341	75	93	92	87	66			
C	2	226	242	204	317	201	344	75	129	106	88				
C	3	193	262	169	318	188	328	79	94	124	91				
C	4	187	241	315	390	198	348	79	117	144	93				
C	5	200	157	122					61	39	81	49			
D	1	200	224	207	334	199	343	69	96	134	116	98			
D	2	206	208	172	309	160	333	82	229	131	116				
D	3	195	215	256	352	198	391	80	78	101	76				
D	4	156	183	253	380	251	394	72	72	92	89				
D	5	206	148	102					57	33	66	51			
E	1	234	159	272			504	67	81	64	96				
E	2	255	261	258			508	80	106	140	107				
F	1	193	201	224			533	79	129	142	102				
F	2	259	236	248			603	77	97	145	105				
Illumina HiSeq 2000 data were provided by 7 sites: BGI (Beijing Genomics Institute), CNL (Weill Cornell Medical College), MAY (Mayo Clinic), AGR (Australian Genome Research Facility), COH (City of Hope), NVS (Novartis), and NYG (the New York Genome Center). Life Technologies SOLiD 5500 data were provided by 4 sites: NWU (Northwestern University), PSU (the Pennsylvania State University), SQW (SeqWright Inc.), and LIV (the University of Liverpool). Roche 454 GS FLX data were provided by: MGP (the Medical Genomes Project), NYU (the New York University Medical Center), and SQW (SeqWright Inc.). For each platform, the first three were official sequencing sites.															
